# News and Coming Events

**Published:** 1908-01-18

**Authors:** 


### NEWS AND COMING EVENTS.

SIR GEORGE BEATSON, K.C.B., M.D., was entertained to dinner in the Grosvenor Banqueting Hall, at Glasgow, on January 10, by a large and representative gathering, under the chairmanship of His Grace the Duke of Montrose. The object of the dinner was to signalise the recent investment of Sir George Beatson with the distinction of Knight Commander of the Bath. The proceedings were most enthusiastic throughout, and the chairman was supported by representatives of the medical profession, the Volunteer Medical Service, the Red Cross Society, the St. Andrew's Ambulance Association, and of the other public organisations with which Sir George has been so long and so prominently associated.

AT a meeting of the Association of Registered Medical Women, held on January 7, the following resolutions were passed :—"That, as women are equally bound with men practitioners by the ethical laws of the profession, women practitioners should, under no circumstances, accept a lower salary than that which has been agreed upon by the profession as a minimum"; "Where men and women practitioners are doing identical work, the rate of payment should be the same." It was stated that under Section 13 of the Education (Administrative Provisions) Act, 1907, it will be the duty of the local education authorities to appoint a number of medical inspectors of schools. It is left to the discretion of the local authorities to appoint men or women to these posts. In the memorandum issued by the Board of Education (circular 576) it is noted that "there are many cases in which women are likely to be specially suitable." It was maintained that, as medical women have had exactly the same training and education as medical men, it was difficult to see on what grounds a different scale of remuneration would be justifiable, as has been suggested in some quarters.

THE London Homœopathic Hospital has received a sum of £10,000 from Sir Henry Tyler towards the £30,000 required for the extension of the hospital on its own freehold site.

THE annual dinner of the Hospital Saturday Fund Association will take place at the Holborn Restaurant on February 1 next, under the presidency of Sir George C. T. Bartley, one of the vice-presidents of the association.

THE ninety-second annual Court of Governors of the Royal Waterloo Hospital for Women and Children will be held at the Mansion House on Friday, January 24 next, at 3 P.M. The Lord Mayor will preside.

THE Lord Chancellor, on the recommendation of the Duke of Fife, K.T., Lord Lieutenant of the County of London, has appointed J. Macdonald Brown, M.D., F.R.C.S., to be a Justice of the Peace for Hampstead.

THE cause list for Hilary Sittings, which opened on Saturday, 11th inst., shows that the General Medical Council is defendant in two actions in the King's Bench Divisional Court. The actions are in the Crown paper, and the nominal prosecutor in each case is Rex. *ex parte* Nicolas.

AT a meeting of the Wantage Board of Guardians, on January 1, Dr. Birt, medical officer at the workhouse, asked the Board to purchase through chemists certain drugs. He stated that during the last two months ointment for scabies had cost him £2 5s., and morphia in another case had cost him £2 5s. The Clerk said that the agreement stipulated that the Medical Officer was to provide drugs. The Board decided to ascertain what practice was adopted by other boards in the district.

